# “I felt like I had been put on the shelf and forgotten about” – lasting lessons about the impact of COVID-19 on people affected by rarer dementias

**DOI:** 10.1186/s12877-023-03992-1

**Published:** 2023-06-27

**Authors:** Emma Harding, Sam Rossi-Harries, Esther Vera Gerritzen, Nikki Zimmerman, Zoe Hoare, Danielle Proctor, Emilie Brotherhood, Sebastian Crutch, Aida Suárez-González

**Affiliations:** 1 Dementia Research Centre, UCL Queen Square Institute of Neurology, UCL, 8-11 Queen Square, London, WC1N 3BG UK; 2 Institute of Mental Health, Mental Health and Clinical Neuroscience, School of Medicine, University of Nottingham, Nottingham, UK; 3 NWORTH Clinical Trials Unit, School of Health Sciences, Bangor University, Bangor, UK; 4 Department of Clinical, Educational, and Health Psychology, UCL Division of Psychology and Language Sciences, UCL, London, UK

**Keywords:** COVID-19, Dementia, Frontotemporal dementia, Primary progressive aphasia, Posterior cortical atrophy, Alzheimer’s disease, Dementia with Lewy bodies

## Abstract

**Background:**

The public health measures imposed in many countries to contain the spread of COVID-19 resulted in significant suspensions in the provision of support and care for people with dementia. The negative effects of these measures have been extensively reported. However, little is known about the specific impact on people with young onset, non-memory-led and inherited dementias. This group may have experienced different challenges compared to those with late onset dementia given their non-memory phenotypes and younger age. We explored the impact of the first COVID-19 lockdown on people living with familial Alzheimer’s disease, behavioural variant frontotemporal dementia, familial frontotemporal dementia, dementia with Lewy bodies, posterior cortical atrophy and primary progressive aphasia and their carers in the UK and their self-reported strategies for coping.

**Methods:**

This was a mixed methods study. An online survey was administered to people with dementia and family carers recruited via Rare Dementia Support. Free-text responses were analysed using framework analysis to identify key issues and themes.

**Results:**

184 carers and 24 people with dementia completed the survey. Overall, people with dementia experienced worsening of cognitive symptoms (70%), ability to do things (62%), well-being (57%) and changes to medication (26%) during lockdown. Carers reported a reduction in the support they received (55%) which impacted their own mental health negatively. Qualitative analysis of free-text responses shed light on how the disruption to routines, changes to roles and responsibilities, and widespread disconnection from friends, family and health and social care support varied according to phenotype. These impacts were exacerbated by a more general sense that precious time was being lost, given the progressive nature of dementia. Despite significant challenges, respondents demonstrated resilience and resourcefulness in reporting unexpected positives and strategies for adapting to confinement.

**Conclusions:**

This study has highlighted the specific impacts of the COVID-19 restrictions on people with young onset, non-memory-led and inherited dementias, including behavioural variant frontotemporal dementia, primary progressive aphasia and posterior cortical atrophy, and their carers. The specific challenges faced according to diagnosis and the self-reported strategies speak to the importance of – and may inform the development of – tailored support for these underrepresented groups more generally.

**Visual abstract:**

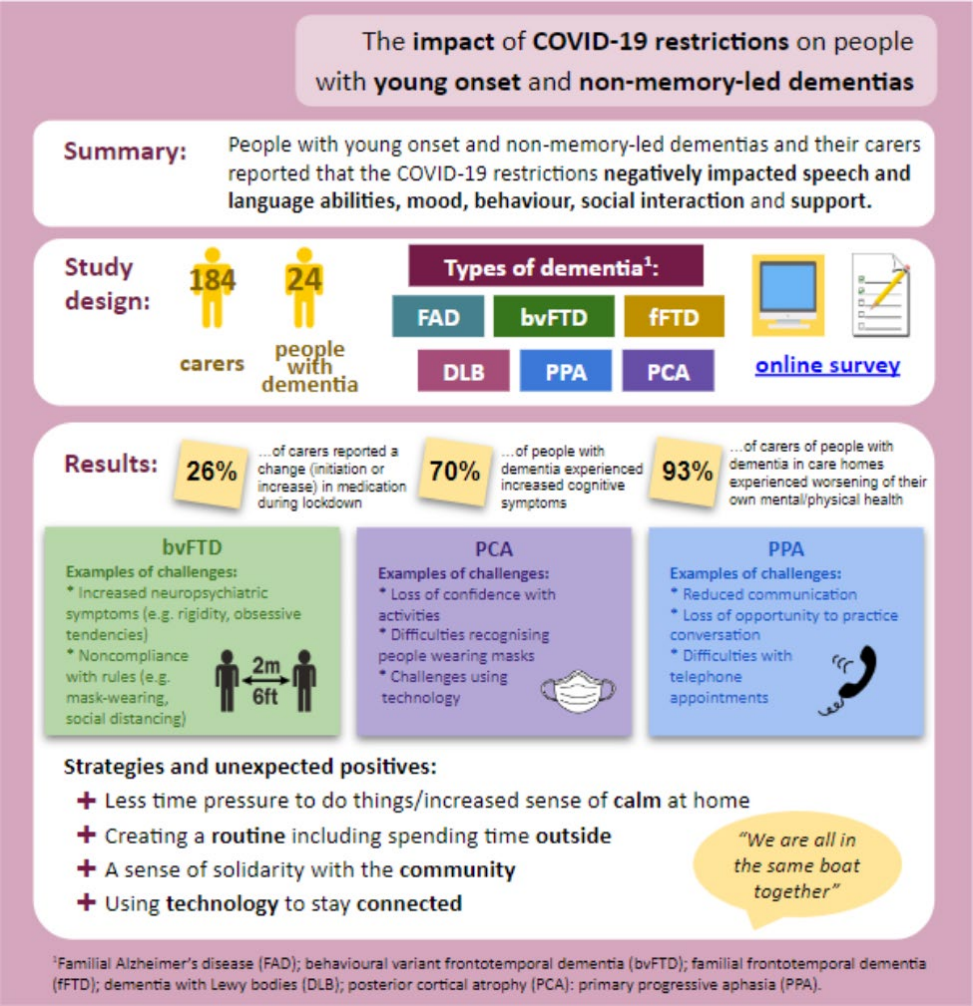

**Supplementary Information:**

The online version contains supplementary material available at 10.1186/s12877-023-03992-1.

## Background

The UK declared a nation-wide lockdown due to the COVID-19 pandemic on 23rd March 2020. The measures that followed involved a stay-at-home order, banning of all non-essential contact with people outside of one’s household and suspension of all non-essential services, leading to a range of negative psychological outcomes for isolated individuals across the globe [1, 2]. Three years on, the significant and detrimental impacts of these restrictions on people living with dementia and their family members have been thoroughly documented [3–10]. The policies to contain COVID-19 impacted many vulnerable groups in society but particularly people living with dementia [11]. Primary sources of support for people with dementia living in the community were removed or significantly reduced throughout the multiple lockdowns [12]. The physical distancing and infection control measures imposed changes to routines, raised barriers for people with dementia to access therapies and health care, and made it more difficult for carers to obtain caring-related support [13]. The main changes reported in people living with dementia under lockdown were worsening of behavioural and psychological symptoms (including apathy, depression, agitation, anxiety and irritability, and walking with purpose), followed by decline in functional abilities for daily living [5, 14, 15] and increased used of antipsychotics [16, 17]. For those in care homes (typically in the later stages of the disease and with more severe symptoms) a decline in cognition and ability to perform activities of daily living, as well as an increase in depression, anxiety, social isolation and responsive behaviours have been reported [18–20].

In contrast, there has been relatively little exploration of the impact of the COVID-19 pandemic and restrictions on those living with young onset, non-memory-led and inherited dementias. The clinical presentation and associated support needs in these dementias differ from the canonical episodic memory decline that characterises typical and sporadic forms of Alzheimer’s disease (AD). People with young onset, non-memory-led and inherited dementias may have faced particular challenges during COVID-19 related not only to their younger age (not fitting within societal perceptions of dementia, e.g., still working), but also the phenotype-specific variation in symptoms [11]. A few studies have highlighted the specific challenges faced by people living with and supporting those with young onset dementias, including frontotemporal dementia (FTD), and the particular challenges related to managing increased behavioural symptoms alongside a multitude of other family care commitments, including caring for young children and/or ageing parents [21, 22]. To build on this, exploration of the impact of COVID-19 of those with other non-memory-led or lesser known dementias such as posterior cortical atrophy (PCA) [23, 24], primary progressive aphasia (PPA) [25], behavioural variant frontotemporal dementia (bvFTD) [26], dementia with Lewy bodies (DLB) [27], and familial forms of both AD (fAD) and FTD (fFTD) [28, 29] is warranted.

The typically earlier age of onset of these forms of dementia results in additional and distinct challenges relating to employment and finances and issues with childcare commitments [30], all of which may have been exacerbated as a result of COVID-19 closures and restrictions. More specifically, the atypical symptoms such as the lack of empathy, apathy and disinhibition that characterise bvFTD, and which is associated with increased carer burden and poorer quality relationships with carers [31], and the communication challenges faced by people with a diagnosis of PPA [32] may have applied additional pressures during enforced household isolation. People with PCA can experience significant impacts on their sense of identity, independence, roles and responsibilities owing to profound difficulties navigating the physical environment [33], another condition-specific feature that may have been accelerated or exacerbated owing to the restricted opportunities for continued engagement in different environments during the COVID-19 restrictions. Those in fAD or fFTD families often face a young onset of symptoms of dementia and additional challenges such as simultaneously occupying multiple roles (e.g. caring for an affected family member while managing their own at-risk status) [28, 29].

Even pre-COVID-19, these challenges had been identified as being exacerbated by the limited awareness about, and consequent lack of tailored support for, young onset and non-memory-led dementias within the health and social care sector [34, 35]. People living with these dementias and their families require specialised care and support [30], and so may have experienced particular challenges during the pandemic. The goal of this study was to describe the lockdown’s impact amongst people affected by young onset, non-memory-led and inherited dementias and their caregivers.

## Methods

The reporting of the methods and results from this survey complies with the CHERRIES survey reporting checklist [36]. This study was conducted as part of the RDS Impact study [37] and granted permission by the UCL Research Ethics Committee (8545/004: RDS Impact Study).

### Study design

A mixed method survey design was chosen to enable cross-comparisons across some areas of known impact across the different diagnostic groups (e.g. whether there was an impact on cognitive functioning, carer mental health) but also to enable unanticipated insights to emerge in the free-text responses about the different experiences across the groups (e.g. the condition-specific nature of the impacts on cognitive functioning, carer mental health). Framework analysis [38]was the approach selected for the qualitative data because it allows for systematic organisation of high volume free-text data relating to individuals’ subjective experience and views of an event or situation, it was developed for use within healthcare contexts and it has previously been applied in studies involving respondents living with dementia and family carers [39, 40].

### Survey development

We built an online survey consisting of 11 questions (one per page), exploring the impact of the lockdown on the person with dementia’s cognitive symptoms, psychological well-being, ability to do things, ability to connect with people socially, general health and changes to medication (see Appendix 1). Questions also explored the health of the caregiver, caring-related support, helpful strategies developed during the lockdown and any positives found in the situation. The first eight questions of the survey could be responded to by selecting either “yes”, “no” or “not sure” (e.g., *Have the person’s cognitive symptoms got worse during the lockdown (for example, being more disoriented, finding it more difficult to communicate).* If respondents marked ‘yes’ in one of the first eight questions, a comment box was provided for the participant to give further details (e.g., *If yes, please tell us how*). The last three questions were open for the respondent to elaborate about (a) helpful strategies to cope with lockdown, (b) positive aspects of the lockdown (if any) and (c) any other additional information about their experience during the lockdown that the respondent wished to share.

The questions were informed by emerging evidence from research in the field [21, 41–43], clinical experience, and feedback from the experience of the Direct Support Team at Rare Dementia Support (RDS) (https://www.raredementiasupport.org/). RDS is an organisation led by the Dementia Research Centre at University College London, which offers education and peer support groups to people with non-memory-led, low prevalence, inherited and young onset dementias. Three variations in the survey questions were produced to suit the three types of respondents approached:

1. People living with dementia
2. Carers of people with dementia living in the community, and
3. Carers of people living in care homes

The electronic questionnaire was built in Qualtrics software version July-August 2020 (Qualtrics, Provo, UT) and tested for technical functionality and usability by three members of the research team (ASG, EH, NZ).

### Recruitment and procedure

The survey was distributed to the RDS mailing list, made up of 1850 members who are either living with or caring for someone with a low prevalence dementia on 11th of August 2020 and remained open until 30th of September 2020. To be included, participants were required to be living with one of the young onset, non-memory-led or inherited forms of dementia supported by RDS, or caring for someone who was, either at home or from a distance. Exclusion criteria was being a professional practitioner member of RDS, rather than a family carer or person living with dementia. RDS members received an email explaining the purpose of the survey (see Appendix 2) and providing instructions for completion along with a link to access the survey. Participants were provided with a link on the survey homepage to the Participant Information Sheet and were required to opt into the survey, confirming this had been read, understood, and the participant was happy to proceed and provide responses. Any member registered in the RDS mailing database, who was either a person living with dementia or a carer, was given the opportunity to participate. This was an open survey since no login was needed to access the survey. However, only RDS members had access to it.

No personal information was collected in this survey, and no incentives were offered for survey completion. A response was not required to proceed to the next page (respondents could progress onwards leaving some questions blank) and respondents were able to go back to check and edit previous entries. Questions were not randomised. The survey was set up so that each respondent could only use the survey link once, and each completed questionnaire was allocated a unique ID. Respondents had to proceed to the end of the survey for their response to be submitted.

### Analysis

The current study presents the quantitative data from the first eight questions, as well as summarised qualitative data from all questions. Responses are expressed in frequency counts (percentages) and contingency tables, and Fisher’s exact test was used for inter-group comparisons. The qualitative responses were analysed using a framework approach [38]. A thematic framework encompassing the different areas of impact (e.g., cognitive function, well-being), phenotype (e.g. bvFTD, PCA) and respondent type (e.g. carer, person with dementia) was created, and individual free-text responses were indexed and charted according to the framework. Key patterns and themes within the responses to each question, as well as key anomalies or differences according to phenotype, were extracted by four members of the research team (ASG, EH, EVG, SRH). Key themes, patterns and illustrative quotes according to each question are descriptively summarised and presented below, and any differences noted according to diagnosis are highlighted.

## Results

### Quantitative results

An email with survey details was sent to 1850 RDS members, including people living with dementia and their carers. We received 208 completed surveys. 184/208 of respondent were caregivers (Table 1) and 24/208 were people living with dementia (Table 2). No statistical differences across diagnosis were found for the survey questions in any of these two groups (p > 0.05, Fisher exact test).

**Table 1:** Responses of carers to the COVID-19 lockdown survey questions

	Total (N = 184)		bvFTD (n = 62)	PCA (n = 59)	PPA (n = 36)	DLB (n = 10)	fFTD (n = 8)	fAD (n = 3)	Other (n = 6)
**Questions about the person with dementia**									
Decline in cognitive symptoms	70%		66%	69%	72%	**80%**	**75%**	**100%**	67%
Negative impact on behaviour or wellbeing	57%		47%	64%	53%	70%	63%	**100%**	**83%**
Loss ability to do things	62%		55%	66%	50%	**90%**	**88%**	67%	**83%**
General health affected negatively	47%		38%	45%	53%	70%	71%	**100%**	67%
Medication changes†	26%		23%	29%	18%	50%	25%	0%	50%
Reduced ability to connect with people socially	71%		60%	**78%**	**75%**	**90%**	**75%**	67%	67%
**Questions about the carer**									
Level of stress or health negatively impacted by the lockdown	**79%**		72%	**80%**	**83%**	**90%**	**75%**	**100%**	**83%**
Negative impact on support received for caring	55%*		52%	58%	58%	50%	40%	**100%**	50%
Harder for carer to provide care and support	93%**		**92%**	**93%**	67%	**100%**	**100%**	**100%**	**100%**

*N = 144, including only carers of people living in the community

**N = 40, including only carers of people living in care homes

† increase or initiation of antidepressants, benzodiazepines, antipsychotics, “sleep medication” (as reported by respondents) and pain killers and in less frequently reported blood thinners, anticholinesterase inhibitors and beta blockers.

**Bold** = 75% or more “yes” responses

Underlined = 50–74% “yes” responses

**Table 2:** Responses of people living with dementia

	Total (N = 24)		bvFTD (n = 5)	PCA (n = 8)	PPA (n = 7)	DLB (n = 3)	fFTD (n = 0)	fAD (n = 1)	Other (n = 0)
Decline in cognitive symptoms	38%		40%	38%	43%	33%	-	0%	-
Negative impact on behaviour or wellbeing	50%		**80%**	38%	29%	**100%**	-	0%	-
Loss ability to do things	46%		60%	38%	43%	67%	-	0%	-
General health affected negatively	42%		40%	38%	29%	**100%**	-	0%	-
Medication changes	13%		0%	0%	14%	33	-	**100%**	-
Negative impact on support received	54%		40%	63%	57%	33	-	**100%**	-
Reduced ability to connect with people socially	74%		40%	**88%**	**83%**	67	-	**100%**	-

**Bold** = 75% or more “yes” responses

Underlined = 50–74% “yes” responses

Overall, 70% of the carers reported a decline in cognitive symptoms and ability to connect with people socially, and a negative impact on their own level of stress and health during the first lockdown in the UK. Medication changes were reported in 26% of the cases.

74% of people with dementia surveyed reported increased difficulties in connecting with people socially due to the lockdown and 50% said that the lockdown had negatively impacted their well-being and the support they received.

#### Carers of people in the community living together

Table 3 summarises responses of carers organised by living situation, including the 120/184 carers who were living together with the person with dementia at the time of completing the survey. 40/120 were carers of people living with PCA, whilst 38 and 25 cared for people with bvFTD and PPA respectively. A minority of respondents were carers of people with DLB, fFTD, fAD and people with other diagnoses (e.g., corticobasal syndrome), or respondents who were unsure about diagnosis. Across all phenotypes, at least 70% of carers reported a decline in the person with dementia’s cognitive symptoms and ability to connect with other people socially during the lockdown, as well as a negative impact on their own health or stress level. The most frequently reported change among carers of people with bvFTD and PPA was a worsening of carers’ health and level of stress (64% and 76% respectively). For carers of people with PCA, it was the loss of the ability of the person with dementia to connect socially (80%). 33% of carers of people with DLB reported changes in medication during the lockdown.

**Table 3:** Responses of carers organised by living situation

**Carers living together in the community with the person with dementia**	**Total** **(N = 120)**	**bvFTD** **(n = 38)**	**PCA** **(n = 40)**		**PPA** **(n = 25)**	**DLB** **(n = 6)**	**fFTD** **(n = 5)**	**fAD** **(n = 2)**	**Other** **(n = 4)**	
**Questions about the person with dementia**										
Decline in cognitive symptoms	70%	61%	**78%**		68%	**83%**	**80%**	**100%**	50%	
Negative impact on behaviour or wellbeing	58%	50%	73%		44%	67%	**80%**	0%	**75%**	
Loss ability to do things	61%	50%	73%		44%	**100%**	**80%**	50%	**75%**	
General health affected negatively	44%	38%	43%		44%	67%	**80%**	0%	50%	
Medication changes	24%	22%	28%		16%	33%	20%	0%	50%	
Reduced ability to connect with people socially	72%	64%	**80%**		68%	**100%**	**80%**	50%	50%	
**Questions about the carer**										
Level of stress or health impacted negatively by the lockdown	73%	68%	73%		**76%**	**83%**	**80%**	**100%**	**75%**	
Negative impact on support received for caring	59%	54%	65%		60%	50%	40%	**100%**	50%	
**Carers of people with dementia living in the community but not together**	**Total** **(N = 24)**	**bvFTD** **(n = 11)**	**PCA** **(n = 5)**		**PPA** **(n = 8)**	**DLB** **(n = 0)**	**fFTD** **(n = 0)**	**fAD** **(n = 0)**	**Other** **(n = 0)**	
**Questions about the person with dementia**										
Decline in cognitive symptoms	63%	64%	40%		**75%**	-	-	-	-	
Negative impact on behaviour or wellbeing	63%	64%	40%		**75%**	-	-	-	-	
Loss ability to do things	71%	73%	**80%**		63%	-	-	-	-	
General health affected negatively	52%	36%	50%		**75%**	-	-	-	-	
Medication changes	29%	36%	20%		25%	-	-	-	-	
Reduced ability to connect socially	**88%**	73%	**100%**		**100%**	-	-	-	-	
**Questions about the carer**										
Level of stress or health impacted by the lockdown	**83%**	64%	**100%**		**100%**	-	-	-	-	
Negative impact on support received for caring	38%	45%	0%		50%	-	-	-	-	
**Carers of people with dementia living in care homes** **Questions about the person with dementia**	**Total** **(N = 40)**	**bvFTD** **(n = 13)**	**PCA** **(n = 14)**	**PPA** **(n = 3)**		**DLB** **(n = 4)**	**fFTD** **(n = 3)**	**FAD** **(n = 1)**	**Other** **(n = 2)**	
Decline in cognitive symptoms	**75%**	**85%**	57%	57%		**75%**	67%	**100%**	**100%**	
Negative impact on behaviour or wellbeing	48%	23%	50%	50%		**75%**	33%	**100%**	**100%**	
Loss ability to do things	60%	54%	43%	43%		**75%**	**100%**	**100%**	**100%**	
General health affected negatively	54%	38%	50%	50%		**75%**	50%	**100%**	**100%**	
Medication changes	30%	15%	36%	36%		**75%**	33%	0%	50%	
Reduced ability to connect socially	60%	38%	64%	64%		**75%**	67%	**100%**	**100%**	
**Questions about the carer**										
Level of stress or health impacted by the lockdown	**93%**	**92%**	**93%**	**93%**		**100%**	67%	**100%**	**100%**	
Harder for carer to provide care and support	**93%**	**92%**	**93%**	**93%**		**100%**	**100%**	**100%**	**100%**	

**Bold** = 75% or more “yes” responses

Underlined = 50–74% “yes” responses

#### Carers of people in the community not living together

Table 3 also summarises the responses of 24/184 carers of people with dementia who did not live together in the same household at the time of survey completion. 11/24 respondents were carers of people with bvFTD, 8 with PPA and 5 with PCA. More than 80% of the carers reported a loss of the ability of the person with dementia to connect with people socially and a worsening of their own stress levels or health. The most frequently reported impact in the bvFTD group was the person with dementia losing the ability to do things and their ability to connect socially. This was similar within the PCA group, in addition to 5/5 carers reporting worsening of their own stress/health levels. All carers of people with PPA reported a loss of ability to connect with other people socially and increased levels of stress/deterioration of caregiver health. 75% of PPA carers also reported a further decline in cognitive symptoms, well-being, behaviour or general health of the person with dementia.

#### Carers of people living in care homes

Table 3 also summarises the responses of 40/184 carers whose relative with dementia was living in a care home at the time of responding to the survey. 93% of carers reported that the lockdown increased their level of stress, and that it became more challenging for them to provide care and support to their relative living in the care home. The majority of respondents were carers of people with bvFTD and PCA. The most commonly reported change in these two groups was a decline in cognition (85%) and the ability to connect with people socially (64%), respectively.

### Qualitative results

**Fig. 1:**
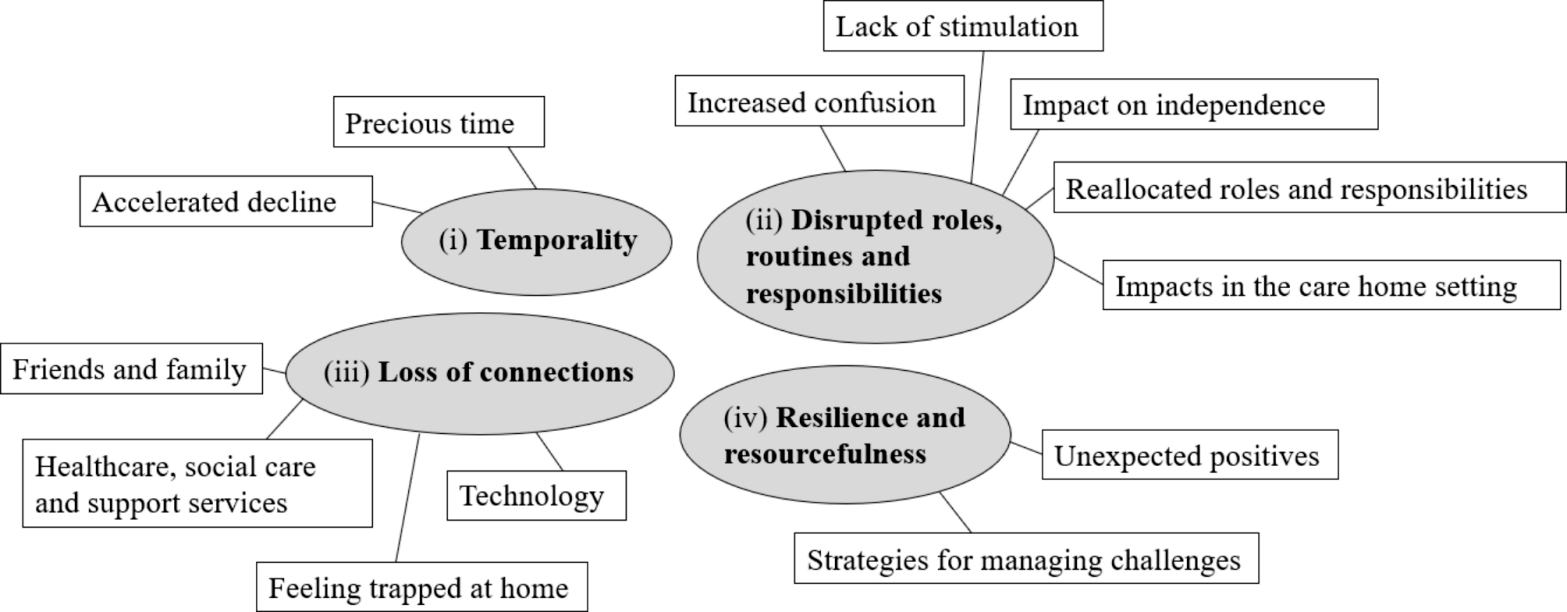
Thematic map of themes and corresponding sub-themes

The qualitative results were organised into four themes which captured the wide-ranging impacts of the COVID-19 restrictions on people with young onset, non-memory-led and inherited dementias and their carers: (i) Temporality: Progressive decline over precious time; (ii) Disrupted roles, routines and responsibilities; (iii) Loss of connections – social connections and connections to services; and (iv) Resilience and resourcefulness. Themes and their corresponding sub-themes are represented in Fig. 1.

### Temporality: Progressive decline over precious time

The first theme captures the importance of the temporal context in shaping people with young onset, non-memory-led and inherited dementia and carers’ experiences of the COVID-19 lockdown restrictions.

#### Accelerated decline

Almost all respondents reported a disproportionate worsening of cognitive, behavioural and/or neuropsychiatric symptoms over time in lockdown compared to previously.

Commonly reported impacts on cognitive abilities along with examples and illustrative quotes are provided in Table 4.

**Table 4:** Commonly reported symptoms, example behavioural observations and quotes

Cognitive symptom	Example behavioural observation	*Illustrative quote*
**Disorientation in time**	Repeatedly asking what time of day it is	*She is now more reliant on dementia clock as she is not always sure, if when her clock says 7.00, if this is morning or evening. Dark or light outside doesn’t act as cue without prompting*
**Disorientation in space**	Not knowing how to get to the bathroom	*Spatial awareness has got much much worse. Finds it nearly impossible to pick things up.*
**Fluctuating awareness**	Seeming engaged one moment and disengaged the next	*Sometimes my mum can talk and function fine - other days just stares into space and doesn’t engage*
**Communication difficulties**	Inability to hold a conversation	*Verbal communication was restricted before lockdown. It is now virtually nonexistent. Has withdrawn more and is less responsive to others.*
**Impaired executive functioning**	Difficulty following instructions to make a cup of tea	*Dressing, showering and cutting meat. Opening cans, following verbal instructions without physical cues and gestures from me i.e. wash your tummy, can’t do unless I pat his tummy to show which bit to wash.*
**Increased difficulty with localizing household objects**	Not being able to find a familiar gardening tool	*Finding objects is very hard. No idea where things are kept in kitchen.*
**Increased difficulty with recognizing/utilizing household objects**	Not knowing how to use a familiar garden tool when handed it	*He no longer recognises everyday tools e.g. garden tools that he would have known how to use before. The language of the word and also the conceptual understanding of everyday objects has deteriorated. He struggles to select knife, fork, spoon etc. by name and no longer recognises shower gel or other toiletries.*

Common behavioural symptoms described by carers of people living with all forms of dementia included increases in agitation, irritability, restlessness, depression, apathy and anxiety. There were also variations within these according to landmark symptoms of the specific phenotype, with several carers in the bvFTD group specifically mentioning increased rigidity and obsessive behaviours (e.g. insisting on walking the same route multiple times) and motor symptoms (e.g. pacing, tapping).

Many also reported additional physical health deterioration alongside this, including reduced mobility, weight changes and increased medication use. For a minority of carers, it was unclear whether the decline they were noticing was to be expected as part of the disease course or whether this was being exacerbated by lockdown. One carer commented:

*My husband seems to get agitated more now, but in all honesty I’m not too sure whether it would be the same, if lockdown had happened or not*

#### Precious time

For many, the sadness of the impacts of the reductions in activity and engagement for the people with young onset, non-memory-led and inherited dementia was amplified by the sense that precious time was being lost because of the progressive nature of the condition. This was especially highlighted by carers of those nearing the end of life. One carer reported:

*I haven’t been able to see in person for 4 months until she is end of life. I only get to see her for 30 mins a day even though she is end of life.*

And another described:

*The emotional stress of this precious time we still have together being taken away from us*

Carers of people living with young onset, non-memory-led and inherited dementia living in the community and those in residential care also expressed concerns about whether or not their relative would have the ability to reengage once restrictions eased, and about the longer-lasting effects of the drastic changes in social contact and activity engagement.

The change over time in the symptoms of the people with dementia was particularly stark for those caring at a distance, with their visits being fewer than previous, meaning they often noticed a greater decline in functioning when they were finally able to see their family member.

### Disrupted routines, roles and responsibilities

The second theme highlights the impacts of the significant disruptions to routines and associated changes in roles that were reported as a result.

#### Increased confusion

Respondents reported that many daily activities and routines were disrupted by the restrictions, including regular contact with family and social groups and activities, and for those living in the community, getting out in the local neighbourhood for walks, shopping, or refreshments, e.g.:

*Before the lockdown I found that if I kept to my routines, I seemed to be able to function better, it may have just been going into the town and meeting up with people for a coffee and a chat, but then with the lockdown that was taken away from me, and I found that I was getting confused more easily.*

Many people with dementia did not understand the reasons for or details of the restrictions. This difficulty was reported as distressing for the people with young onset, non-memory-led and inherited dementia, and also challenging for carers who mentioned repeatedly trying to explain the restrictions, and occasionally having to manage the people with young onset, non-memory-led and inherited dementia’s noncompliance with mask wearing or social distancing, as well as others’ reactions to this, e.g.:

*I still take her out shopping, though only to shops where we are known, and they are happy to allow her in with no mask.*

*However, his friends have stopped visiting possibly because of COVID and his ability to adhere to health guidelines, social distance etc.*

#### Lack of stimulation

Changes in routines were often characterised by a reduction in activity engagement for people with young onset, non-memory-led and inherited dementia and consequent lack of stimulation. This was reported as challenging by people with young onset, non-memory-led and inherited dementia and those supporting them. People with PCA and PPA reported a loss of confidence in engaging with activities following this period of reduced stimulation, for example:

*I firmly believe that the lockdown situation has had this catastrophic effect on my mum’s ill health mostly attributed to lack of social contact and stimulation from visitors which in turn led to her refusing food/swallowing issues causing dramatic weight loss which made her weak and unable to weight bear and whilst I acknowledge that she would have reached this stage eventually I do not think it would have been so rapid*

They also reported feelings of boredom as a result of less engagement, e.g.:

*Due to Covid restrictions they have reduced outside activities (outings, walks etc). As dad is still very physically active this compounded his transition as he is bored and frustrated.*

Many carers attributed the increase in some behavioural symptoms described above to this reduction in stimulation, e.g.:

*My husband has become more restless, I think partly due to all activities and variety that had been built into his week disappearing.*

#### Impact on independence

Roles and responsibilities within the household changed as a result of declined independence. People with young onset, non-memory-led and inherited dementia were reported as having more difficulty independently performing activities including food and drink preparation, personal care (e.g. dressing and bathing), household chores (e.g. laundry, washing dishes, shopping), engaging with enjoyable activities (e.g. gardening and puzzles, navigating around the local area), and using technology. These activities appeared to be disrupted in different ways for the different diagnostic groups. For example, navigating to and around the local shops for someone with PCA was made more difficult because of the challenges of facial recognition exacerbated by mask-wearing, with one carer commenting:

*Higher level of anxiety e.g., more anxious going into public settings. Facial recognition is hard enough with PCA but face masks exacerbate the issue*

#### Reallocated roles and responsibilities

These disrupted activities and reduced independence often meant increased roles and responsibilities for carers of those people with young onset, non-memory-led and inherited dementias living at home (e.g. extra hands-on assistance for washing), which was reported as being extremely stressful and overwhelming by many. Carers also commonly reported secondary stresses including feelings of guilt about the frustration and impatience they were experiencing.

#### Impacts in the care home setting

Carers of people living in care homes reported feelings of great sadness and guilt at being unable to meet their relative’s physical, emotional and social needs, as they usually would due to the ban on visits. One daughter explained:

*I find speaking to her on the phone and via video call quite distressing and it can also be difficult to arrange with the care home, especially full time working from home and parenting. This means that I’m not in contact as often as I want to be.*

Carers described missing physical contact with their relatives, especially when their relative was non-verbal or approaching the end of life. Even once visits were allowed, they were for the most part described as difficult to organize due to staff shortages and limited correspondence. Carers also reported that the required personal protective equipment, time limits and being restricted to meeting outside meant visits did not feel the same or provide carers or people with young onset, non-memory-led and inherited dementias with the connection, comfort and reassurance they relied on visits for, for example:

*Loss of access to my wife has been stressful, as our main communication is by touch - she cannot speak. Video calls, “window” visits and more recently 2m distant visits by the care home entrance do not always elicit a response from her.*

When not visiting, carers expressed concerns regarding the level of stimulation their relative was receiving, and there were many fears reported about the person with dementia being isolated in their room. Worries about their relative’s health and well-being were exacerbated by having to rely on reports from care home staff who were under incredible amounts of pressure:

I would have hoped that hospitals would have improved their communication with relatives when visits were not allowed, but this was not the case and was a source of stress and anxiety. I would have hoped that they would have made an exception for the relatives of people with dementia to visit their loved ones, but they didn’t, and I think this inability to see my sister/her to see us, contributed to the decline in her dementia and in her physical state.

### Loss of connections – social connections and connections to services

This theme reflects the profound sense of a loss of connection and the impacts on people with dementia and carers.

#### Friends and family

Reduced visits from family and friends were reported as greatly missed, and restricted access to social spaces within the neighbourhood (e.g., cafes and churches) was another key way in which social connectedness had reduced.

People with PPA in particular reported experiencing a loss of ability to practice conversational skills with others as a result of restrictions.

#### Healthcare, social care and support services

Health care appointments with a professional involved in the clinical care of the people with young onset, non-memory-led and inherited dementia were disrupted, including appointments with the GP, neurologist, psychiatrist, speech and language therapists and occupational therapy. Disruptions included cancellations, increased waiting times and migration to online or telephone appointments. In a minority of cases, the reduction in services had contributed to the development of medical complications. One person with dementia shared:

*I only have phone consultations from the psychiatrist which is difficult because of the effect of my PPA, so my husband helped but I still found it stressful. Also, the same with my GP […]. I’m struggling with symptoms but I’m avoiding the phone consult.*

Social care services were also cancelled or moved online, including: home care, companions, sitting service, and day centres, and support from third sector organisations, including support groups (for carers and people with dementia) and activity groups (e.g., Singing for Dementia, Men in Sheds). Many carers and people with young onset, non-memory-led and inherited dementia reported feelings of isolation and a lack of social connection as a result of these changes with knock-on impacts for psychological well-being, e.g.:

*No dementia social groups means no networking with other carers. No carers support groups to let off steam.*

#### Technology

Difficulties using technology posed significant barriers to maintaining social connectedness throughout the lockdown. The nature of these barriers varied by diagnosis. The visual symptoms characteristic of PCA were reported as making engaging with technological interfaces difficult. One person shared:

*Zoom is difficult for me with my visual difficulties and I get frustrated that I can’t talk directly to people.*

People with PPA reported experiencing particular difficulties with speaking on the phone:

*No or little contact with family or friends initially and then only an increased level of phone and video contact. Not good for someone with PPA*

#### Feeling trapped at home

Many carers reported feeling trapped at home without their usual respite, e.g.:

*No face-to-face group activities/carers meetings. Some support online but still feel ‘stuck’ at home!*

This too could be exacerbated for different reasons across the different diagnostic groups.

For some carers of people with PCA this feeling of being trapped tended to result from increased ‘clinginess’ of those with PCA who were experiencing heightened anxiety levels. For carers of people with PPA, this similar sense of feeling trapped at home was more often exacerbated by the lack of opportunities for communication with their relative owing to their speech and language problems. Feelings of being trapped in the group of carers of people with bvFTD were reported as driven by the stresses associated with increased time spent at home dealing with exacerbated neuropsychiatric symptoms with no opportunity for respite. Some carers of people with bvFTD also described feelings of isolation resulting from their relative not appreciating the extra demands they were facing.

### Resilience and resourcefulness

This theme reflects the resilience and resourcefulness respondents demonstrated in adapting to the challenges arising from the restrictions. Participants reported a wealth of strategies they had found helpful for managing the many challenges resulting from the COVID-19 restrictions, as well as unexpected positives which arose from the restrictions. These are summarised in Table 5.

**Table 5:** Strategies and unexpected positive reported by respondents

**Strategies for coping with/managing the impact of COVID-19 restrictions**	
	**General:**
	Increased use of technology for maintaining existing connections and establishing new ones, e.g.: *“Family Zoom meetings have now become a regular event and will probably continue after Covid”* *“I have managed to join Zoom groups with people that live further away so I would not normally attend their meetings as I’m not in the correct area”*
	Imposing routine, regular outdoor activity (e.g. walks in green spaces)
	Engaging with meaningful and accessible activities within the home, e.g. evoking memories of the past by using photographs and memory books
	Conflict avoidance strategies, e.g. steering conversation away from difficult subjects
	**bvFTD-specific strategies**:
	Maintaining a calm and peaceful home environment
	Engaging activities such as puzzles, jigsaws, colouring and sticker books
	Hiding food and drink as a helpful strategy for minimising over-consumption
	Making the local community aware of the person´s diagnosis so they could understand the struggles to comply with restrictions
	**PCA-specific strategies**:
	Engaging with home-based activities which maximise other senses than vision, e.g. listening to podcasts or the radio
	**PPA-specific strategies**:
	Engaging with activities which minimise the demands on language and communication skills such as crafting and gardening
**Unexpected positives arising from COVID-19 restrictions**	
	Increased flexibility for carers’ with their work patterns, e.g. more opportunity for working remotely
	Increased opportunities to nurture caring relationships due to decreased external demands on time
	Increased opportunities for spending time on hobbies, creative activities and other restorative activities such as exercise
	Being introduced to new technologies that helped with managing care demands, e.g. online grocery shopping
	Having less to do increased the sense of calm within the home environment by reducing the levels of stress and anxiety that usually accompanied leaving the house and having to get to appointments or activities on time, e.g.: *“her mental health has improved, with isolation and no contact from anyone except carers (therefore less change and less confusion)”*
	A beneficial sense of camaraderie, along with a sense that the wider public were now more able to empathise with their situation, due to their own isolation, e.g.: *“her mental health has improved, with […] the sense of camaraderie of everyone suffering the disorientating effects of the covid lockdown, my mother in law has become happier more stable and much more coherent on the phone than before […] we think it is the fact that it is not just her who is suffering her changed perception of the world - all she hears on the radio is people talking about coping with a new reality the new reality now means we are all in the same boat together”*
	Appreciation for the local community as a result of the compassion, kindness and support shown in response to the pandemic and restrictions

## Discussion

This study aimed to explore the impacts of COVID-19 restrictions on people affected by young onset, non-memory-led and inherited dementias and their family carers across domains including cognitive functioning, behaviour and wellbeing, ability to do things, carer mental health and social connectedness. The results showed that the majority of carers and people with dementia reported negative impacts on social connection, the person with dementia’s ability to do things, access to and (for carers) ability to provide support, cognitive functioning, and carer health and wellbeing as a result of the COVID-19 restrictions. Analysis of free-text responses highlighted additional challenges with navigating these impacts in the context of a progressive condition, the manifold ways roles, routines and responsibilities were disrupted, but also the resilience and resourcefulness many people affected by rarer dementias and family carers demonstrated in response.

The majority of respondents in this study were carers of people with bvFTD, PCA and PPA and their free-text responses shed light on the specific challenges posed by the COVID-19 restrictions according to the different phenotypes [24–26]. These included: increased rigidity, obsessive behaviours and a lack of appreciation of the increase in caring demands reported by carers of people with bvFTD; increased difficulty with facial recognition due to mask wearing and with navigating technological interfaces like the Zoom app reported by carers of people with PCA; and difficulties with participating in telephone consultations and a loss of valued opportunities to practice and maintain confidence in speaking for people with PPA. Our results are in keeping with other studies describing the impact of lockdown on people living with bvFTD and DLB, finding that 62% of people with DLB and 55% with bvFTD experienced the onset or worsening of psychological and behavioural symptoms [43]. Altogether, the detrimental effects of lockdown on the cognitive, mental health and functional abilities of people with dementia and on the mental and physical health of carers reported in our study are in line with other recent studies. A recent rapid systematic review of one year of quantitative evidence concluded that the effects of COVID-19 isolation measures include worsening of cognition and worsening or new onset of behavioural and psychological symptoms for people with dementia or mild cognitive impairment [14]. Another study highlighted the particular challenges people caring from a distance faced with coping with restrictions to visiting their family member with dementia [44]. Another study’s findings also illustrated the lack of access and changes in delivery formats for support, and the challenges of managing the impacts of the pandemic restrictions alongside the deterioration characteristic of dementia [45]. The barriers to engaging with digital technologies for people with dementia and the concerns about the longer-term impacts of prolonged social disconnection reported here have also been documented in other studies. One study demonstrated the range of potential barriers to engaging with services remotely, including device or platform-related limitations which impacted the quality of social connection as identified in this study, for example the challenges of managing audio delays and the potential loss of a sense of connection when asking participants to mute their microphones [46]. Another study captured the complex layers of loneliness resulting from the loss of social connection, like those identified here, including people with dementia missing contact with their peers with a shared experience, and carers feeling a sense of loneliness due to a loss of conversation and communication with the person with dementia (due to their cognitive difficulties) alongside a sense of aloneness in holding the responsibility for keeping the person with dementia engaged and stimulated [47]. Previous studies addressing the impact of dementia services closures and disruptions within the UK during the COVID-19 pandemic have also yielded results that support our findings including: the significant emotional impacts of reduced access to respite [10], concerns about the deterioration of the person with dementia and their ability to reengage in services once they reopen [12], the direct impact of this loss of services and support on the wellbeing of people with dementia and carers [13, 47, 48], the challenges for people with dementia to understand the restrictions and reasons for closures [49], and the importance of the relationship and contact arrangements with care homes for the psychological wellbeing of carers [49]. Our findings about changes to medication – reported in 26% of cases across the groups – are in keeping with other recent studies and typically also related to commencement or increase in the use of antipsychotics, benzodiazepines and antidepressants [42, 43].

In accordance with previous studies, even in the face of severe adversity some respondents were able to develop helpful strategies and experience some positives during lockdown including carers having more time to allocate to their caring responsibilities [48], the introduction to digital technologies with potential to support social connection and daily activities (e.g. shopping) [50], the benefits of a simplified routine [51], and increased opportunities for quality time with the person with dementia as well as self-care for carers [52].

### Implications

#### Acknowledging diagnostic diversity

This study highlighted the significant negative impacts of the COVID-19 restrictions on people with low prevalence and non-memory-led dementias, and in the case of many of these diagnoses, for the first time. We have identified numerous ways in which often the same restrictions and responses to them (e.g., social withdrawal, mask-wearing, use of technology), can manifest and be experienced in different ways according to these different diagnoses. This highlights the need for tailored and specialised support for people affected by these atypical conditions, both at times of crisis such as the COVID-19 pandemic and as we continue to settle in to a ‘new normal’.

#### Recognising resilience

This study has also highlighted the resourcefulness of those affected by young onset, non-memory-led and inherited dementias and their carers in showcasing the many creative and tailored adaptations and strategies they developed to cope during the COVID-19 crisis. This builds on previous studies in drawing attention to the wealth of expertise-by-experience and resilience those facing these challenges in their everyday lives possess [50–52], while adding nuance in terms of identifying particular strategies which may be helpful for those with particular diagnoses. Identifying beneficial approaches that facilitate resilience and the navigation of daily life during lockdown may be of relevance to support these populations during future pandemics and indeed in other times of extreme stress or disruption, particularly when people may be confined to the home (e.g., due to ill health) or without their usual access to support.

#### Moving forward

Acknowledging the unique needs and skills of those affected by young onset, non-memory-led and inherited dementias will be particularly important as we fully adjust to the relaxing of restrictions, particularly given how many respondents expressed their concerns about a new set of challenges that will come with adjusting to this ‘new normal’, in this study and others, and the long-term negative psychological health outcomes identified for those who have undergone quarantine and isolation globally [1, 2, 5, 6, 53]. The adaptability respondents have shown in making use of technology to support communication and social connection, in this study and others, holds promise for the development of innovative approaches to remote health care and social interaction in the future [45, 54, 55]. Adaptations such as these may have additional benefits for those with low prevalence dementias who are more likely to be geographically spread and less likely to come into contact with others familiar with their condition within local services. As indicated in the challenges outlined above, the provision of adequate support which is sensitive to varying profiles of cognitive impairment will be imperative in maximizing digital accessibility and engagement.

### Recommendations for health and social care practitioners

- Make sure to ask clients and/or family carers for the subtype of dementia diagnosis (if known) to enable the delivery of appropriate and tailored support and intervention.
- Be alert to any non-memory symptoms the person with dementia is experiencing.
- Acknowledge any creative adaptations and resourcefulness demonstrated by clients and families affected by dementia to foster empowerment and resilience.
- Encourage and signpost people affected by young onset, non-memory-led and inherited dementias to age and condition appropriate peer support to enhance experience-sharing, encourage swapping of strategies and to reduce isolation.
- Probe and problem solve around any barriers to digital inclusion in a person-centred way, to take account of any relevant variations in cognitive phenotypes and to improve access to digital sources of support.

#### Limitations

Some methodological limitations should be acknowledged. First, no demographics were collected in this survey, which impedes comparisons across groups. On a similar note, due to the pragmatic and responsive nature of the survey, it was not possible to make comparisons with a control group. Second, limitations also apply to the representativeness of this sample. For instance, it may be that people who felt more stressed were more motivated to share their experience and therefore, may be overrepresented. In contrast, it is also possible that people who felt more stressed were less available and are therefore underrepresented. Third, only people able to use technology could engage in the online survey. This is an important limitation for many, with deeper repercussions in the representativeness of data collected online, but also equity and equality in accessing research and other services. Fourth, people with dementia were also encouraged to participate in the survey, however, no system was in place to support their participation, which biases both who could respond to the survey and the responses themselves. For instance, only 38% of people with dementia reported changes in cognitive status, while 70% of carers did. This may be because only people in mild stages of the disease were able to engage in the survey or due to poorer awareness of the cognitive changes experienced. Barriers to people with dementia engaging and participating digitally remain a big challenge. Fifth, the interpretability of informant-reported data on clinical symptoms must be treated cautiously, especially in the cases of lesser-known dementias where carers themselves may have a limited understanding of what particular cognitive difficulties underpin the everyday challenges they are witnessing in their relative.

## Conclusions

Social and cognitive stimulation, specialised therapeutic support and respite for carers are essential to enable people to live well with dementia [56, 57], yet these fundamental pillars of the dementia care pathway were significantly interrupted during the COVID-19 pandemic. This paper has documented the specific impacts of the COVID-19 lockdown restrictions on people living with young onset and non-memory led dementias including behavioural variant frontotemporal dementia, dementia with Lewy bodies, posterior cortical atrophy, and primary progressive aphasia, and their family members, so that (i) their unique needs are considered within national responses to future times of crisis and disruption, and (ii) lessons can be learned from their resilience and resourcefulness in responding to the COVID-19 pandemic.

## Electronic supplementary material

Below is the link to the electronic supplementary material.

Appendix 1. Survey.

Appendix 2. Email sent to RDS members explaining the purpose of the survey and providing instructions for completion.

## Data Availability

The data that support the findings of this study are available on request from the corresponding author, [ASG]. The data are not publicly available in compliance with ethic and funding requirements but will be uploaded to a data repository for researchers from different institutions to access after the study ends in 2024.

## List of Abbreviations

bvFTD: Behavioural variant frontotemporal dementia
DLB: Dementia with Lewy bodies
FAD: Familial Alzheimer’s disease
fFTD: Familial frontotemporal dementia
PCA: Posterior cortical atrophy
PPA: Primary progressive aphasia
