# Supplementary material for: “I felt like I had been put on the shelf and forgotten about” – lasting lessons about the impact of COVID-19 on people affected by rarer dementias
Source: BMC Geriatr. 2023 Jun 27;23:392. doi: 10.1186/s12877-023-03992-1 (PMC10303850; doi:10.1186/s12877-023-03992-1)
Supplement: Supplementary file 1 — Appendix 1. Survey. [file 12877_2023_3992_MOESM1_ESM.docx]

**APPENDIX 1.** Survey

___________________________________

COVID-19 Impact on people affected by rare dementias

Start of Block: Intro to survey

**Understanding the impact of COVID-19 on people affected by rare dementias - RDS Impact Study**
 
We are aware that the current COVID-19 situation is posing particular challenges for people affected by rarer dementias. As part of the Rare Dementia Support (RDS) Impact Study, we invite you to share your experiences with us in this brief survey.  
 
Although we will ask for some information about your situation, please do not give any information that might identify another person in your responses - for example instead of using another person's name, please use a general term (e.g. instead of saying "I've noticed that Frank...", say, "I've noticed that my husband...").
 

We are aware that reflecting on and writing about any difficult experiences may be distressing and would encourage you to contact our Direct Support Team at contact@raredementiasupport.org if you would like to talk to somebody about any of the issues raised here.
 
This survey is part of the RDS Impact Study, ethical approval for which has been granted by the UCL Research Ethics Committee. You can read the full information sheet for the study by clicking [here](https://drive.google.com/file/d/123_XSkPyxgZ92zsqutiFvkHIbxyzg_vY/view).

Do you confirm that you have read and understood the information provided about this study and are happy to complete this online questionnaire?

- Yes, I wish to proceed (1)
- No, I do not wish to proceed (2)

Skip To: End of Survey If Do you confirm that you have read and understood the information provided about this study and ar... != Yes, I wish to proceed

End of Block: Intro to survey

Start of Block: PLWD/Carer

Please let us know which of these best describes you:

- I have a diagnosis of a dementia (1)
- I am supporting someone with a diagnosis of a dementia (2)

End of Block: PLWD/Carer

SURVEY VARIANT 1: PEOPLE LIVING WITH DEMENTIA

Start of Block: Confirming basic info (PLWD)

Please let us know which type of dementia you have:

- Familial Alzheimer's disease (FAD) (1)
- Familial Frontotemporal dementia (fFTD) (2)
- Frontotemporal dementia (FTD) (7)
- Lewy Body Dementia (3)
- Posterior Cortical Atrophy (PCA) (4)
- Primary Progressive Aphasia (PPA) (5)
- Other (please specify): (6) ________________________________________________

End of Block: Confirming basic info (PLWD)

Start of Block: PLWD questions re: impact

This section is about the impacts of the COVID-19 situation on you.

Do you think that your **cognitive symptoms** have got worse during lockdown?

- Yes (1)
- No (2)
- Not sure (4)

If yes, please tell us how:

________________________________________________________________

________________________________________________________________

________________________________________________________________

________________________________________________________________

________________________________________________________________

| Page Break |  |
| --- | --- |

Has lockdown had a negative impact on your **wellbeing**?

- Yes (4)
- No (5)
- Not sure (7)

 If so, please tell us how:

________________________________________________________________

________________________________________________________________

________________________________________________________________

________________________________________________________________

________________________________________________________________

| Page Break |  |
| --- | --- |

Are you finding it more difficult **to do things** than you were before the lockdown?

- Yes (4)
- No (5)
- Not sure (7)

If so, what things have you been finding more difficult?

________________________________________________________________

________________________________________________________________

________________________________________________________________

________________________________________________________________

________________________________________________________________

| Page Break |  |
| --- | --- |

Has the lockdown negatively affected your **physical health** at all?

- Yes (4)
- No (5)
- Not sure (7)

If yes, please tell us how:

________________________________________________________________

________________________________________________________________

________________________________________________________________

________________________________________________________________

________________________________________________________________

| Page Break |  |
| --- | --- |

Q90 Have you required changes in your medication as a consequence of the lockdown?

- Yes (1)
- No (2)
- Not sure (3)

Q91 If so, in what ways has your medication changed?

________________________________________________________________

________________________________________________________________

________________________________________________________________

________________________________________________________________

________________________________________________________________

| Page Break |  |
| --- | --- |

Has the COVID-19 crisis negatively affected the **support you receive**?

- Yes (4)
- No (5)
- Not sure (7)

If yes, please tell us in which ways:

________________________________________________________________

________________________________________________________________

________________________________________________________________

________________________________________________________________

________________________________________________________________

| Page Break |  |
| --- | --- |

Has lockdown had a negative impact on your ability to **connect with people socially**?

- Yes (6)
- No (7)
- Not sure (8)

If so, please tell us how:

________________________________________________________________

________________________________________________________________

________________________________________________________________

________________________________________________________________

________________________________________________________________

End of Block: PLWD questions re: impact

Start of Block: Coping strategies (PLWD)

Are there any **strategies you have found helpful** during confinement?

________________________________________________________________

________________________________________________________________

________________________________________________________________

________________________________________________________________

________________________________________________________________

While we know the COVID-19 crisis has come with many challenges, we would also like to know if, in contrast, there have been any positives to be found in the situation at all, so any aspects of daily life which perhaps haven't been as negatively impacted as expected or which have even improved during this time. If so, please tell us about them here:

________________________________________________________________

________________________________________________________________

________________________________________________________________

________________________________________________________________

________________________________________________________________

Is there anything else you would like to tell us about how the COVID-19 situation has impacted you?

________________________________________________________________

________________________________________________________________

________________________________________________________________

________________________________________________________________

________________________________________________________________

End of Block: Coping strategies (PLWD)

Start of Block: RDS service options

This survey is distributed by **Rare Dementia Support (RDS)**. RDS is a world-leading, UK-based service led by the UCL Dementia Research Centre (DRC). We aim to empower, guide and inform people living with a rare dementia diagnosis and those who care about them. To find out more about the 6 rare dementias we support and to become a member please visit our website [here](https://www.raredementiasupport.org/). 
 

End of Block: RDS service options

SURVEY VARIANT: CARERS PEOPLE LIVING WITH DEMENTIA

Start of Block: Confirming basic info (carer)

Please let us know which type of dementia the person you are supporting has:

- Familial Alzheimer's disease (FAD) (1)
- Familial Frontotemporal dementia (fFTD) (2)
- Frontotemporal dementia (FTD) (7)
- Lewy Body dementia (3)
- Posterior Cortical Atrophy (PCA) (4)
- Primary Progressive Aphasia (PPA) (5)
- Other (please specify): (6) ________________________________________________

Please let us know about the living situation of the person you are supporting:

- The person I am supporting lives with me at home in the community (1)
- The person I am supporting lives at home in the community but not with me (2)
- The person I am supporting lives in a residential or nursing care home (3)

End of Block: Confirming basic info (carer)

SURVEY VARIANT 2: CARERS PEOPLE WITH DEMENTIA LIVING IN CARE HOMES

Start of Block: Carer care home questions re: impact

This section is about the impacts of the COVID-19 situation on you and the person with dementia you support. We appreciate it may be difficult to answer some of these questions if you have had restricted access to your relative - it is completely fine to skip any questions you don't feel able to answer.

Have the person's cognitive symptoms got worse during the lockdown (for example being more disoriented, finding it more difficult to communicate ...)?

- Yes (1)
- No (2)
- Not sure (4)

If yes, please tell us how:

________________________________________________________________

________________________________________________________________

________________________________________________________________

________________________________________________________________

________________________________________________________________

| Page Break |  |
| --- | --- |

Has the COVID-19 situation had a negative impact on your relative's behaviour or psychological wellbeing, for instance, showing more agitation, apathy, depression etc.?

- Yes (4)
- No (5)
- Not sure (7)

 If so, please tell us about them:

________________________________________________________________

________________________________________________________________

________________________________________________________________

________________________________________________________________

________________________________________________________________

| Page Break |  |
| --- | --- |

Has your relative lost the ability **to do things** that they could do before the lockdown?

- Yes (4)
- No (5)
- Not sure (7)

If so, what things have they lost the ability to do?

________________________________________________________________

________________________________________________________________

________________________________________________________________

________________________________________________________________

________________________________________________________________

| Page Break |  |
| --- | --- |

Has the lockdown had a negative impact on the the **general health** of your relative with dementia?

- Yes (4)
- No (5)
- Not sure (7)

If yes, please tell us how. For example, have they lost weight, experienced physical decline, developed additional health issues, etc. during this time?

________________________________________________________________

________________________________________________________________

________________________________________________________________

________________________________________________________________

________________________________________________________________

| Page Break |  |
| --- | --- |

Has your relative required changes in **medication**as a consequence of the lockdown?

- Yes (4)
- No (5)
- Not sure (7)

If so, in what ways has their medication changed?

________________________________________________________________

________________________________________________________________

________________________________________________________________

________________________________________________________________

________________________________________________________________

| Page Break |  |
| --- | --- |

Has your level of **stress** or your **health** (either physical or psychological) been negatively impacted by the lockdown?

- Yes (1)
- No (2)
- Not sure (4)

If so, please tell us how:

________________________________________________________________

________________________________________________________________

________________________________________________________________

________________________________________________________________

________________________________________________________________

| Page Break |  |
| --- | --- |

Has the COVID-19 crisis made it harder for you to provide **care and support** for your relative?

- Yes (4)
- No (5)
- Not sure (7)

If yes, please tell us in which ways and how this has affected you both:

________________________________________________________________

________________________________________________________________

________________________________________________________________

________________________________________________________________

________________________________________________________________

| Page Break |  |
| --- | --- |

Q92 Has lockdown had a negative impact on your relative's ability to connect with people socially?

- Yes (1)
- No (2)
- Not sure/Not applicable (3)

Q93 If so, please tell us how:

________________________________________________________________

________________________________________________________________

________________________________________________________________

________________________________________________________________

________________________________________________________________

End of Block: Carer care home questions re: impact

Start of Block: Coping strategies (carer care home)

Are there any **strategies you have found helpful** for coping with the lockdown?

________________________________________________________________

________________________________________________________________

________________________________________________________________

________________________________________________________________

________________________________________________________________

While we know the COVID-19 crisis has come with many challenges, we would also like to know if, in contrast, there have been any positives to be found in the situation at all, so any aspects of daily life which perhaps haven't been as negatively impacted as expected or which have even improved during this time. If so, please tell us about them here:

________________________________________________________________

________________________________________________________________

________________________________________________________________

________________________________________________________________

________________________________________________________________

Is there anything else you would like to tell us about how the COVID-19 situation has impacted you and your relative with dementia?

________________________________________________________________

________________________________________________________________

________________________________________________________________

________________________________________________________________

________________________________________________________________

End of Block: Coping strategies (carer care home)

SURVEY VARIANT 3: CARERS PEOPLE WITH DEMENTIA LIVING IN THE COMMUNITY

Start of Block: Carer in community questions re: impact

This section is about the impacts of the COVID-19 situation on you and the person with dementia you support.

Have the person's cognitive symptoms got worse during the lockdown (for example being more disoriented, finding it more difficult to communicate ...)?

- Yes (1)
- No (2)
- Not sure (4)

If yes, please tell us how:

________________________________________________________________

________________________________________________________________

________________________________________________________________

________________________________________________________________

________________________________________________________________

| Page Break |  |
| --- | --- |

Has the COVID-19 situation had a negative impact on your relative's **behaviour or psychological wellbeing**, for instance, showing more agitation, apathy, depression etc.?

- Yes (4)
- No (5)
- Not sure (7)

 If so, please tell us about them:

________________________________________________________________

________________________________________________________________

________________________________________________________________

________________________________________________________________

________________________________________________________________

| Page Break |  |
| --- | --- |

Has your relative lost the ability **to do things** that they could do before the lockdown?

- Yes (4)
- No (5)
- Not sure (7)

If so, what things have they lost the ability to do?

________________________________________________________________

________________________________________________________________

________________________________________________________________

________________________________________________________________

________________________________________________________________

| Page Break |  |
| --- | --- |

Has the lockdown negatively affected the **general health** of the person you are supporting?

- Yes (4)
- No (5)
- Not sure (7)

If yes, please tell us how. For example, have they lost weight, experienced physical decline, developed additional health issues, etc. during this time?

________________________________________________________________

________________________________________________________________

________________________________________________________________

________________________________________________________________

________________________________________________________________

| Page Break |  |
| --- | --- |

Has your relative required changes in **medication**as a consequence of the lockdown?

- Yes (4)
- No (5)
- Not sure (7)

If so, in what ways has their medication changed?

________________________________________________________________

________________________________________________________________

________________________________________________________________

________________________________________________________________

________________________________________________________________

| Page Break |  |
| --- | --- |

Has your level of **stress** or your **health** (either physical or psychological) been negatively impacted by the lockdown?

- Yes (1)
- No (2)
- Not sure (4)

If so, please tell us how:

________________________________________________________________

________________________________________________________________

________________________________________________________________

________________________________________________________________

________________________________________________________________

| Page Break |  |
| --- | --- |

Has COVID-19 negatively affected the **support** you receive in caring?

- Yes (4)
- No (5)
- Not sure (7)

If yes, please tell us more about how:

________________________________________________________________

________________________________________________________________

________________________________________________________________

________________________________________________________________

________________________________________________________________

| Page Break |  |
| --- | --- |

Q88 Has lockdown had a negative impact on your relative's ability to connect with people socially?

- Yes (6)
- No (7)
- Not sure/Not applicable (8)

Q89 If so, please tell us how:

________________________________________________________________

________________________________________________________________

________________________________________________________________

________________________________________________________________

________________________________________________________________

End of Block: Carer in community questions re: impact

Start of Block: Coping strategies (carer)

Are there any **strategies you have found helpful** to support your relative during confinement?

________________________________________________________________

________________________________________________________________

________________________________________________________________

________________________________________________________________

________________________________________________________________

While we know the COVID-19 crisis has come with many challenges, we would also like to know if, in contrast, there have been **any positives** to be found in the situation at all, so any aspects of daily life which perhaps haven't been as negatively impacted as expected or which have even improved during this time. If so, please tell us about them here:

________________________________________________________________

________________________________________________________________

________________________________________________________________

________________________________________________________________

________________________________________________________________

Is there anything else you would like to tell us about how the COVID-19 situation has impacted you and your relative with dementia?

________________________________________________________________

________________________________________________________________

________________________________________________________________

________________________________________________________________

________________________________________________________________

End of Block: Coping strategies (carer)
