# Supplementary material for: “I felt like I had been put on the shelf and forgotten about” – lasting lessons about the impact of COVID-19 on people affected by rarer dementias
Source: BMC Geriatr. 2023 Jun 27;23:392. doi: 10.1186/s12877-023-03992-1 (PMC10303850; doi:10.1186/s12877-023-03992-1)
Supplement: Supplementary file 2 — Appendix 2. Email sent to RDS members explaining the purpose of the survey and providing instructions for completion. [file 12877_2023_3992_MOESM2_ESM.docx]

**APPENDIX 2.** Email sent to RDS members explaining the purpose of the survey and providing instructions for completion.

-------------------------------------------------------------------------------------------------------------------------------------

**Dear RDS members**

We appreciate that this continues to be a very difficult time for many of our members, and recently published [this letter in the British Medical Journal](https://eur01.safelinks.protection.outlook.com/?url=https%3A%2F%2Fwww.bmj.com%2Fcontent%2F369%2Fbmj.m2489&data=04%7C01%7C%7C2cef2c7998404d20fcce08d89daabf0a%7C1faf88fea9984c5b93c9210a11d9a5c2%7C0%7C0%7C637432703961995437%7CUnknown%7CTWFpbGZsb3d8eyJWIjoiMC4wLjAwMDAiLCJQIjoiV2luMzIiLCJBTiI6Ik1haWwiLCJXVCI6Mn0%3D%7C1000&sdata=fvfmQ27XunSv4iqTQtmPFuWCuOSODoCg5J6K2me0Xxc%3D&reserved=0) to draw attention to the specific needs of people with non-memory-led dementias at this time. To continue raising awareness and ensuring that the needs and rights of people with rare dementias are on the agenda of policy-makers, communities and institutions, we are looking to capture and disseminate evidence about the specific impact of the pandemic on RDS members. We would like to know more about your experiences of lockdown, the many and varied ways it has impacted you and your loved ones, any ways you have found to respond and manage, as well as any unexpected positives to be taken from the significant ways our lives have all suddenly altered.

We have designed a brief survey and we would greatly appreciate anything you are able to share about your experience here: [**SURVEY - Impact of COVID-19 on people with rare dementias**](https://uclpsych.eu.qualtrics.com/jfe/form/SV_etUprFxMbYcHyId). The survey is open to people living with a diagnosis of a rare dementia and those caring for or supporting someone with a rare dementia, whether they are at home or in residential care.

We are really grateful for anything you are able to share, but also mindful that many of you are facing significant challenges day-to-day and may not be able to – we would like to remind you that our Direct Support Team are available for one-to-one support and can be contacted at: [contact@raredementiasupport.org](mailto:contact@raredementiasupport.org)

With very best wishes,

*The Rare Dementia Support Team.*
